# Regulation of autonomic nervous system by acupuncture: a heart rate variability study on physical stress

**DOI:** 10.3389/fnhum.2025.1676863

**Published:** 2025-11-11

**Authors:** Lun Li, Sha Liang, Jinfeng Bai, Yun Zeng, Mengzhen Zhang, Zhongwen Li, Dingshang Yan, Yangming Hu, Liang He, Yizhe Liu, Qi Liu, Yingjun Zhang, Min Feng

**Affiliations:** 1 School of Traditional Chinese Medicine, Hunan University of Medicine, Huaihua, China; 2 School of Rehabilitation Medicine and Health, Hunan University of Medicine, Huaihua, China; 3 College of Acupuncture-Moxibustion and Tuina, Shaanxi University of Chinese Medicine, Xian, China; 4 Department of Medical Imaging, Hunan University of Medicine, Huaihua, China

**Keywords:** acupuncture, moxibustion, ST36, CV12, autonomic nervous system, physical stress, heart rate variability

## Abstract

**Introduction:**

This study aimed to investigate the effects of acupuncture and moxibustion, as well as various acupoints, on human autonomic nervous system (ANS) function and physical stress. The research further to identify effective intervention strategies for stress management and health maintenance.

**Methods:**

A Self-comparison design was conducted with healthy volunteers. Thirty-five volunteers received sequential 15-min interventions of moxibustion at ST36, acupuncture at CV12, and acupuncture at ST36, with a one-day washout period between interventions. Heart rate variability (HRV) was measured to assess autonomic function, and heart rate (HR) and the physical stress index (PSI) were measured to assess stress levels.

**Results:**

Compared with the baseline, acupuncture at ST36 increased the high-frequency power (HF), root mean square of the successive interval difference (RMSSD), instantaneous standard deviation of the R–R interval (SD1), long-term standard deviation of the R–R intervals (SD2), total power (TP), and standard deviation of the normal–normal interval (SDNN). Acupuncture at CV12 increased RMSSD, SD1, SD2, TP and SDNN, with sustained effects for RMSSD and SD1 post-acupuncture, SD1/SD2 were increased post-acupuncture. The HRs of all three interventions decreased during the intervention, and remained sustained effects post-intervention. The PSI decreased during acupuncture and the stimulation at CV12 remained sustained effects post-acupuncture.

**Conclusion:**

Acupuncture alleviates physical stress by regulating ANS activity, with distinct modulatory effects observed across different acupoints, indicating potential applications in stress management and health maintenance. Moxibustion demonstrates marked efficacy in reducing HR.

**Clinical trial registration:**

https://itmctr.ccebtcm.org.cn/mgt/search, ITMCTR2025001289.

## 1 Introduction

Chronic stress has become a widespread problem affecting the physical and mental health of individuals of all ages (Schleinzer et al., 2024). Stress serves as the adaptive system of the body in response to external stimuli. However, when stress exceeds the body’s regulatory capacity, it can lead to adverse physiological and psychological responses, potentially impacting lifespan (Agorastos and Chrousos, 2022). Alleviating excessive stress is a critical factor in maintaining overall health and preventing the development of various chronic diseases, including hypertension, stroke, and obesity (Hill et al., 2022).

The stress response of an organism is orchestrated by neural, endocrine, and immune mechanisms, with principal mediation by the ANS and the hypothalamic–pituitary–adrenal (HPA) axis. Upon stressor perception, sympathetic nervous system activation triggers neurotransmitter and hormone release, which in turn drives both subsequent biochemical and behavioral changes (Gosain et al., 2020). The ANS maintains physiologic equilibrium through dynamic sympathetic–parasympathetic antagonism, and low levels of parasympathetic activity are associated with stress (Goreis et al., 2023). Stress also produces objective physiologic changes in the body, such as changes in HRV (Arakaki et al., 2023). HRV refers to the physiological phenomenon characterized by fluctuations in the time intervals between consecutive heartbeats (R–R intervals on an electrocardiogram). HRV reflects subtle changes in both time and frequency within each sinus heart cycle and can be used to describe the efferent activity of the sympathetic and parasympathetic branches of the ANS (Khan et al., 2019). High levels of HRV are positively correlated with health (Arakaki et al., 2023); conversely, chronic hyperstress states exhibit pathognomonic HRV suppression and reduced parasympathetic nervous system activity (Thielmann et al., 2021). There are different indicators of HRV, and it is generally accepted that HF, RMSSD, and SD1 reflect vagal function; LF and SD2 reflect sympathetic function; TP, SDNN reflects the overall activity of the ANS; and SD1/SD2 reflect the balance between the sympathetic and parasympathetic nervous systems (Shaffer and Ginsberg, 2017). Elevated stress levels in the body can manifest as a decrease in HF and an increase in LF in HRV (Goreis et al., 2023). The PSI, which is based on HRV data, is used to comprehensively assess the physiological and psychological stress experienced by the body (Pluntke et al., 2019; Kim et al., 2018).

Acupuncture and moxibustion are commonly used external treatment methods in traditional Chinese medicine (TCM) and are widely used throughout the world (Yan et al., 2022). Acupuncture involves stimulating acupoints on the body with needles, whereas moxibustion involves burning moxa wool or other non-moxa materials to apply heat to acupoints or affected areas (Wang and yue, Z., 2021). In addition to treating disease, acupuncture and moxibustion are often used for health maintenance (Birch et al., 2020; You et al., 2021). Previous studies have shown that acupuncture can reduce self-perceived stress levels (May and Bennett, 2023), prevent test anxiety (Fleckenstein et al., 2018), and improve stress-related depressive symptoms (Guo et al., 2024). Acupoints are the main stimulation points in acupuncture and moxibustion therapy, and in TCM theory, different acupoints may have similar effects. For example, both Zusanli (ST36) and Zhongwan (CV12) are recognized for their health-promoting properties. Therefore, the choice of treatment method and acupuncture point is a crucial factor in determining treatment efficacy. An increasing number of acupuncture studies employ HRV monitoring to assess the effects of acupuncture on ANS activity. Meta-analysis results indicate that acupuncture treatment significantly modulates HRV more effectively than placebo, thereby enhancing physical and mental well-being (Hamvas et al., 2023). However, how acupuncture and moxibustion, as well as different acupoints, affect the real-time regulation characteristics of the ANS and their intervention effects on stress are currently unclear.

Based on the foregoing, we propose the hypothesis that “acupuncture alleviates physical stress by regulating ANS activity.” Using HRV to assess ANS activity and stress levels, this study aimed to assess real-time changes in ANS activity in healthy young individuals by acupuncture and moxibustion at the same acupoint (ST36) and acupuncture at different acupoints (ST36, CV12), investigate the effects of acupuncture and moxibustion, as well as various acupoints, on human ANS function and physical stress. The research further to identify effective interventions for stress management and health maintenance.

## 2 Materials and methods

### 2.1 Study design

**Figure 1:**
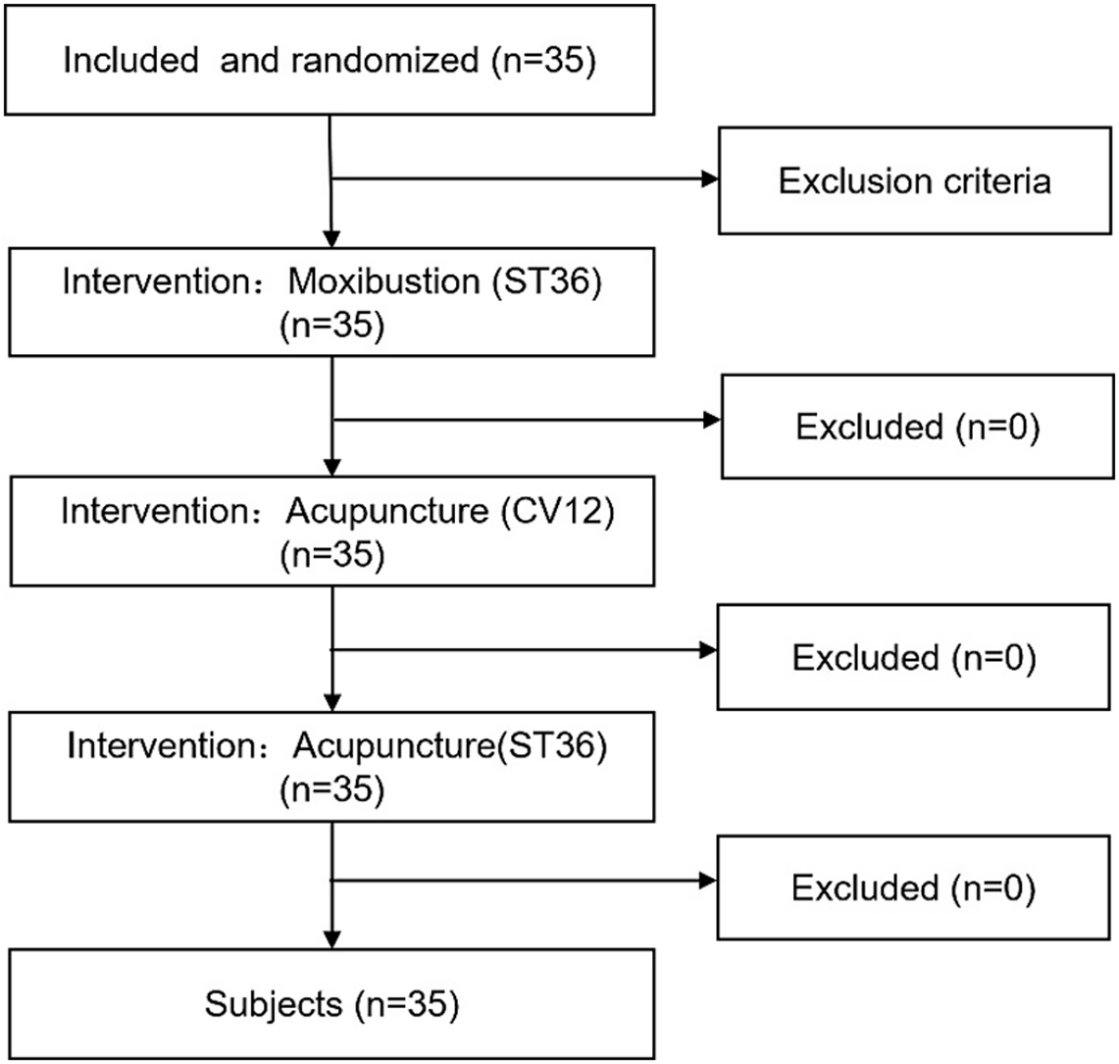
Study flow chart.

A Self-comparison design was conducted and a pilot study (*n* = 15) was carried out among healthy young individuals prior to the main study. On the basis of the pre-experimental SDNN data, sample size calculations were performed. The mean difference in the SDNN between acupuncture at ST36 and moxibustion at ST36 was 5 ms, with a significance level of 0.05, a power of 0.9, and a potential drop-out rate of 0.1, the required sample size was determined that to be 35 participants. The participants were recruited from Hunan University of Medicine. We randomized the order of therapies. The participants underwent three interventions in sequence: moxibustion at ST36, acupuncture at ST36 and acupuncture at CV12, with a one-day washout period between interventions. During the study, the participants were in the supine position. The procedures were rigorously performed by 5 acupuncturists who underwent 1 week of standardized training and evaluation according to the protocol. Blinding was implemented for both HRV data entry personnel and result evaluators. The study flow chart is shown in Figure 1. This study was conducted in accordance with the tenets of the Declaration of Helsinki, and the protocol was approved by the Ethics Committee of Hunan University of Medicine, approval number: 2022 (H01001). Clinical Trial Registration Number: ITMCTR2025001289. All the participants volunteered and signed an informed consent form.

### 2.2 Participants

The inclusion criteria were as follows: (1) healthy adults aged 18–25 years; (2) no history of heart disease, hypertension, diabetes, neurological disorders, psychological disorders, chronic pain conditions (women without dysmenorrhea in the last 3 months) or obesity; and (3) willing to participate in the study and signing an informed consent form.

The exclusion criteria were as follows: (1) pregnant, planning to become pregnant, or breastfeeding; (2) bleeding tendency; or (3) suspected disease.

The elimination criteria were as follows: (1) use of any medication affecting HR within 24 h prior to the study; (2) skin breakage at the site of operation; (3) individuals with arrhythmia or distorted ECG recordings; (4) individuals who felt pain only during acupuncture; (5) individuals who did not feel the needle sensation; or (6) automatically excluded individuals if records were incomplete.

### 2.3 Interventions

#### 2.3.1 Acupoint localization

**Figure 2:**
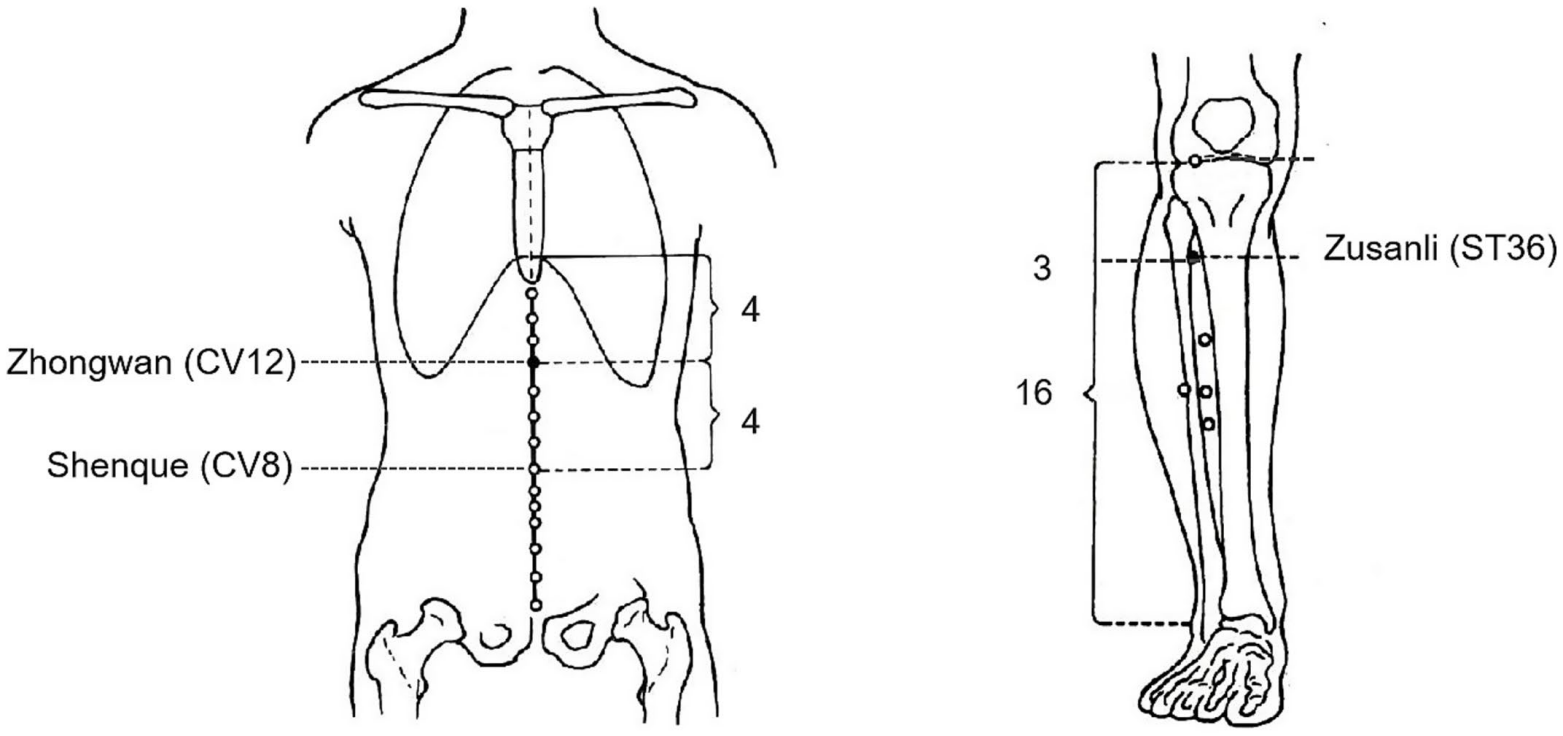
Anatomical localization of the Zhongwan (CV12) and the Zusanli (ST36).

“Zhongwan” (CV12) is located at the midpoint between the xiphosternal synchondrosis and the umbilicus, 4 cun (body-specific unit in TCM) above the umbilicus. “Zusanli” (ST36) is located 3 cun below Dubi (ST35), on the line connecting Dubi (ST35) and Jiexi (ST41) (see Figure 2).

#### 2.3.2 Acupuncture methods

**Figure 3:**
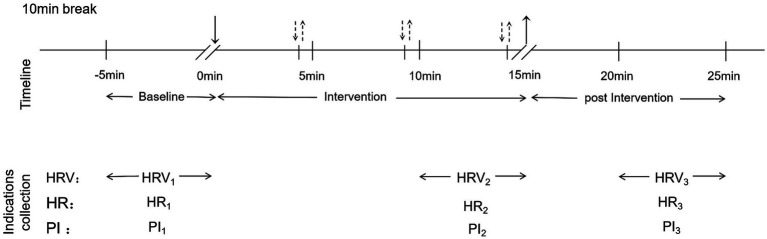
Single intervention timeline. ↓: start of intervention; ↑: end of intervention; ⇣⇡: reinforcement-reduction method (acupuncture).

(1) Acupuncture method: participants were positioned in the supine position with the upper abdomen or right lower leg exposed. Local disinfection was performed with 75% alcohol. A tube needle (0.30 × 40 mm, Huatuo, Suzhou, China) was inserted 0.8–1.2 cun deep at the Zhongwan acupoint or Zusanli acupoint and held for 15 min. Reinforcement–reduction methods were performed every 5 min (uniform lifting and thrusting combined with twirling and rotation at a frequency of 60 times per minute, lasting for 30 s), while ensuring that the needling sensation intensity remained between 2 and 5 scores (Massachusetts General Hospital Acupuncture Sensation Scale, MASS).
(2) Moxibustion method: participants were placed in the supine position, the right lower leg was exposed, and 75% alcohol was used for local disinfection. Mild moxibustion was performed on the Zusanli acupoint using a moxibustion box for 15 min (the distance between the moxa stick and the skin was adjusted on the basis of the participant’s sensation, with the criteria being that the participants feel comfortable and that the local skin turn slightly red after moxibustion) (see Figure 3).

### 2.4 Metrics

#### 2.4.1 Heart rate (HR) and physical stress index (PSI)

HR was obtained from the ECG recordings. The PSI was used to determine stress levels and assess physical adaptation and was calculated by the Kubios HRV version 3.1.0 software on the basis of ECG data.

#### 2.4.2 Heart rate variability (HRV)

The short-term 5-min recording method was employed (Perulli et al., 2023). The participants were instructed to ensure adequate sleep and to refrain from vigorous exercise, alcohol, strong tea and coffee the night before the test. Electrocardiogram data were collected via an HR monitor pre-intervention, during the 10–15 min of the intervention, and 5–10 min post-intervention. The participants lay down for 10 min before collection, and the supine position was used throughout the test. During ECG acquisition, limb movement was avoided, the environment was kept quiet, and the room temperature was maintained at 25 ± 1 °C. Parameters such as SDNN and RMSSD for time domain analysis, TP, LF and HF for frequency domain analysis, and SD1, SD2 and SD1/SD2 for non-linear measures were selected to assess overall ANS activity, vagal activity, sympathetic activity and sympathetic-vagal balance.

### 2.5 Data processing

The double independent entry method was used. Kubios HRV version 3.1.0 (Kubios Oy: Kuopio, Finland) was used for HRV parameters analysis and SPSS 26.0 was used for data analysis. Repeated measures analysis of variance (RM-ANOVA) was used to assess main effect of time, a value of *p* < 0.05 was considered to indicate a statistically significant difference. Post-hoc pairwise comparisons between different intervention were performed using Bonferroni correction, and a value of *p* < 0.0167 was considered to indicate a statistically significant difference.

## 3 Results

### 3.1 Participants

Thirty-five participants (see Table 1) completed all three tests, and all the data were collected.

**Table 1:** Participant demographics.

N	Age (year)	Sex	
		Female	Male
35	19.3 ± 0.5	20	15

### 3.2 Indicators of vagal activity

Compared with the baseline, HF, RMSSD and SD1 (*p* = 0.004, d = 0.53; *p* < 0.001, *d* = 0.68; *p* < 0.001, *d* = 0.68) increased during acupuncture at ST36 and no statistically significant differences were detected post-acupuncture (*p* = 0.123, *p* = 0.122, *p* = 0.122). Compared with that during acupuncture, RMSSD and SD1 decreased post-acupuncture (*p* < 0.001, *d* = 0.69; *p* < 0.001, *d* = 0.69).

Compared with the baseline, RMSSD and SD1 (*p* = 0.002, *d* = 0.55, *p* = 0.002, *d* = 0.55) increased during acupuncture at CV12 and remained elevated post-acupuncture (*p* = 0.001, *d* = 0.55; *p* = 0.001, *d* = 0.60). Compared with that during acupuncture, no statistically significant differences were detected in RMSSD and SD1 (*p* = 0.856, *p* = 0.853). No statistically significant differences were detected in HF at baseline, during acupuncture, or post-acupuncture at CV12 (*p* = 0.101).

No statistically significant differences were detected in HF, RMSSD, or SD1 at baseline, during moxibustion, or post-moxibustion at ST36 (*p* = 0.070, *p* = 0.207, *p* = 0.205) (see Table 2).

**Table 2:** Comparison of baseline, during intervention, and post-intervention measurements across indicators of HRV for three interventions.

Indicator	Intervention methods	Baseline	During intervention	Post-intervention	Tests of Within-subjects effects	*p*-value (Cohen’s d)		
						Pre-Mid	Pre-Post	Mid-Post
HF (ms^2^)	A (CV12)	1291.17 ± 1216.29	1521.57 ± 1362.78	1458.06 ± 1313.83	0.101			
	A (ST36)	935.31 ± 687.32	1342.31 ± 1075.01	1088.66 ± 844.83	0.002^##^	0.004^a^ (0.53)	0.123	0.017
	M (ST36)	1830.14 ± 2191.51	1556.57 ± 1563.86	1640.69 ± 1906.73	0.070			
RMSSD (ms)	A (CV12)	52.65 ± 27.86	59.19 ± 30.33	58.86 ± 31.01	0.001^##^	0.002^a^ (0.55)	0.001^a^ (0.55)	0.856
	A (ST36)	47.59 ± 21.10	58.72 ± 27.58	50.98 ± 22.86	0.000^##^	0.000^a^ (0.68)	0.122	0.000^a^ (0.69)
	M (ST36)	60.06 ± 35.67	57.63 ± 31.44	60.49 ± 35.25	0.207			
SD1 (ms)	A (CV12)	37.29 ± 19.74	41.91 ± 21.48	41.68 ± 21.97	0.001^##^	0.002^a^ (0.55)	0.001^a^ (0.60)	0.853
	A (ST36)	33.70 ± 14.95	41.59 ± 19.53	36.10 ± 16.20	0.000^##^	0.000^a^ (0.68)	0.122	0.000^a^ (0.69)
	M (ST36)	42.54 ± 25.27	40.81 ± 22.28	42.85 ± 24.97	0.205			
LF (ms^2^)	A (CV12)	918.00 ± 631.32	1054.43 ± 765.89	1100.51 ± 839.09	0.085			
	A (ST36)	819.17 ± 613.39	1098.80 ± 721.90	1055.00 ± 982.52	0.091			
	M (ST36)	892.57 ± 751.28	1067.71 ± 1053.86	981.43 ± 692.93	0.381			
SD2 (ms)	A (CV12)	73.23 ± 23.31	86.32 ± 27.89	76.69 ± 25.94	0.000^##^	0.000^a^ (0.91)	0.172	0.006^a^ (0.51)
	A (ST36)	68.04 ± 23.13	88.27 ± 30.25	75.69 ± 26.04	0.000^##^	0.000^a^ (0.97)	0.043	0.002^a^ (0.56)
	M (ST36)	75.96 ± 29.14	80.57 ± 31.22	80.26 ± 26.04	0.439			
TP (ms^2^)	A (CV12)	3272.43 ± 1997.40	4196.06 ± 2525.26	3672.29 ± 2244.27	0.000^##^	0.000^a^(0.73)	0.027	0.022
	A (ST36)	2881.03 ± 1791.39	4177.17 ± 2494.50	3500.94 ± 2142.83	0.002^##^	0.000^a^ (0.68)	0.055	0.044
	M (ST36)	3937.46 ± 3358.60	4094.60 ± 3074.28	4263.46 ± 3316.82	0.729			
SDNN (ms)	A (CV12)	58.58 ± 20.36	68.37 ± 23.44	62.29 ± 22.48	0.000^##^	0.000^a^ (0.88)	0.036	0.012^a^ (0.45)
	A (ST36)	54.01 ± 18.49	69.52 ± 24.04	59.79 ± 20.33	0.000^##^	0.000^a^ (0.99)	0.034	0.001^a^ (0.64)
	M (ST36)	62.14 ± 25.92	64.50 ± 25.48	65.04 ± 31.37	0.439			
SD1/SD2	A (CV12)	0.49 ± 0.16	0.47 ± 0.15	0.53 ± 0.18	0.006^##^	0.158	0.045	0.003^a^ (0.53)
	A (ST36)	0.49 ± 0.16	0.46 ± 0.16	0.47 ± 0.17	0.438			
	M (ST36)	0.53 ± 0.19	0.50 ± 0.19	0.52 ± 0.19	0.270			
HR (bpm)	A (CV12)	69.09 ± 8.97	65.29 ± 8.38	66.43 ± 7.81	0.001^##^	0.000^a^ (1.30)	0.000^a^ (0.87)	0.004^a^ (0.52)
	A (ST36)	71.86 ± 9.35	68.20 ± 9.18	69.31 ± 9.32	0.000^##^	0.000^a^ (0.95)	0.000^a^ (0.70)	0.029
	M (ST36)	68.29 ± 10.66	65.74 ± 8.77	65.51 ± 8.66	0.000^##^	0.000^a^ (0.66)	0.000^a^ (0.67)	0.547
PSI	A (CV12)	11.18 ± 7.32	10.07 ± 6.698	10.87 ± 6.99	0.001^##^	0.001^a^ (0.60)	0.004^a^ (0.53)	0.979
	A (ST36)	11.33 ± 5.83	10.20 ± 6.38	10.59 ± 5.68	0.006^##^	0.007^a^ (0.48)	0.027	0.213
	M (ST36)	10.87 ± 6.99	10.94 ± 7.78	10.29 ± 6.80	0.319			

The “Tests of Within-Subjects Effects” column reports the statistics for the main effect of time in repeated measures ANOVA (^##^*p* < 0.01); For the same intervention method (within-row comparisons), a indicates significant differences in pairwise post-hoc comparisons after Bonferroni correction (corrected *p* < 0.0167). Cohen’s d values were calculated for pairs showing significant differences.

The results suggest that acupuncture can elevate indicators related to vagal activity in real time and the stimulation at CV12 maintains effects for 5–10 min post-acupuncture.

### 3.3 Indicators of sympathetic activity

Compared with the baseline, SD2 (*p* < 0.001, *d* = 0.97) increased during acupuncture at ST36 and no statistically significant differences were detected post-acupuncture (*p* = 0.043). Compared with that during acupuncture, SD2 decreased post-acupuncture (*p* = 0.002, *d* = 0.56). No statistically significant differences were detected in LF at baseline, during acupuncture, or post-acupuncture at ST36 (*p* = 0.091).

Compared with the baseline, SD2 (*p* < 0.001, *d* = 0.91) increased during acupuncture at CV12 and no statistically significant differences were detected post-acupuncture (*p* = 0.172). Compared with that during acupuncture, SD2 decreased post-acupuncture (*p* = 0.006, *d* = 0.51). No statistically significant differences were detected in LF at baseline, during acupuncture, or post-acupuncture at CV12 (*p* = 0.085).

No statistically significant differences were detected in LF or SD2 at baseline, during moxibustion, or post-moxibustion at ST36 (*p* = 0.381, *p* = 0.439) (see Table 2).

The results suggest that acupuncture can elevate indicators related to sympathetic activity in real time.

### 3.4 Total ANS power and sympathetic-vagal balance

Compared with the baseline, TP and SDNN (*p* < 0.001, *d* = 0.068; *p* < 0.001, *d* = 0.99) increased during acupuncture at ST36 and no statistically significant differences were detected post-acupuncture (*p* = 0.055, *p* = 0.034). Compared with that during acupuncture, no statistically significant differences were detected in TP (*p* = 0.044). SDNN (*p* = 0.001, *d* = 0.64) decreased post-acupuncture. No statistically significant differences were detected in SD1/SD2 post-acupuncture (*p* = 0.438).

Compared with the baseline, TP and SDNN (*p* < 0.001, *d* = 0.073; *p* < 0.001, *d* = 0.88) increased during acupuncture at CV12 and no statistically significant differences were detected post-acupuncture (*p* = 0.027, *p* = 0.036). Compared with that during acupuncture, no statistically significant differences were detected in TP post-acupuncture (*p* = 0.022). SDNN (*p* = 0.012, *d* = 0.45) decreased and SD1/SD (*p* = 0.003, *d* = 0.53) increased post-acupuncture.

No statistically significant differences were detected in TP, SDNN or SD1/SD2 at baseline, during moxibustion, or post-moxibustion at ST36 (*p* = 0.729, *p* = 0.439, *p* = 0.270) (see Table 2).

The results suggest that acupuncture can elevate indicators related to total ANS power in real time and acupuncture at CV12 can elevate SD1/SD2 reflecting sympathetic-vagal balance at 5–10 min post-acupuncture.

### 3.5 HR and PSI

Compared with the baseline, HR and PSI (*p* < 0.001, *d* = 0.95; *p* = 0.007, *d* = 0.48) decreased during acupuncture at ST36. HR (*p* < 0.001, *d* = 0.70) remained lowered and no statistically significant differences were detected in PSI post-acupuncture (*p* = 0.027). Compared with that during acupuncture, no statistically significant differences were detected in HR or PSI post-acupuncture. (*p* = 0.029, *p* = 0.213).

Compared with the baseline, HR and PSI (*p* < 0.001, *d* = 1.3; *p* = 0.001, *d* = 0.60) decreased during acupuncture at CV12 and remained lowered post-acupuncture (*p* < 0.001, *d* = 0.87; *p* = 0.004, *d* = 0.53). Compared with that during acupuncture, HR (*p* = 0.004, *d* = 0.52) increased and no statistically significant differences were detected in PSI post-acupuncture (*p* = 0.979).

Compared with the baseline, HR (*p* < 0.001, *d* = 0.66) decreased and remained lowered post-moxibustion (*p* < 0.001, *d* = 0.67). Compared with that during moxibustion, no statistically significant differences were detected in HR post-moxibustion (*p* = 0.547). No statistically significant differences were detected in PSI at baseline, during moxibustion, or post-moxibustion at ST36 (*p* = 0.319) (see Table 2).

The results suggest that all three interventions can decrease HR, with effects sustained for 5–10 min post-intervention. Acupuncture can decrease PSI, and stimulation at CV12 maintains effects for 5–10 min post-acupuncture.

## 4 Discussion

The results of this study show that acupuncture can activates the global activity of the vagus nerve, sympathetic nervous system and ANS in real time. Acupuncture at CV12 continued to enhance vagal activity for 5–10 min post-acupuncture, facilitating a shift in autonomic balance toward vagal dominance. Both acupuncture and moxibustion reduced HR, with effects sustained for 5–10 min post-intervention. Acupuncture shows efficacy for reducing physical stress with effects sustained at CV12.

This study selected CV12 and ST36 due to their established roles in TCM as health-preserving points, with clinical evidence supporting their efficacy in stress relief. Given the body’s single CV12 acupoint, unilateral stimulation is also employed for ST36. Research indicates comparable therapeutic efficacy between left and right lower extremity stimulation (Wang et al., 2010). Refer to similar studies, we uniformly selected the right ST36 point (Jie et al., 2018). Five acupuncturists performed all acupuncture procedures using tube needles to standardize needle insertion technique. A 1-day washout period was implemented based on pilot study data to minimize cross-therapy interference with HRV measurements and standardize administration timing. The formal experimental results also demonstrated that a 1-day washout period could eliminate the effects of single acupuncture and moxibustion sessions on human HRV. Our study participants were healthy young adults, specifically university students facing academic stress, making them suitable observational subjects. The sample exhibited a balanced gender ratio with good representativeness, and the high homogeneity in age and background helped avoid confounding effects of factors such as age, disease, and lifestyle on HRV.

In recent years, several studies have confirmed acupuncture’s ability to modulate ANS function (Fan, 2023; Meira do Valle and Hong, 2024), although there are differences in the regulation of specific nerve branches (Fu et al., 2024; Yang et al., 2024). Taken together, these findings underscore the therapeutic potential of acupuncture in autonomic regulation. Unfortunately, our current results did not indicate changes in ANS activity caused by moxibustion at ST36, a finding consistent with previous HRV studies in qi-deficient populations (Song et al., 2020). This discrepancy in efficacy is likely due to mechanistic differences between acupuncture and moxibustion. The immediate effects of acupuncture are largely dependent on the nervous system. The immediate effects of acupuncture are largely dependent on the nervous system (Qu et al., 2024; Kouzuma et al., 2022) and are rapid in onset. Our previous analgesia research demonstrated ANS modulation within the first 5 min of treatment (Liu et al., 2024). In contrast, moxibustion generates heat and radiation on acupoints or diseased areas by burning moxa wool, primarily altering blood composition and hemorheology, and regulating vascular dilation and constriction functions (Lu et al., 2023). It requires a certain amount of time for cumulative thermal for therapeutic efficacy. The duration of moxibustion is an important component of moxibustion dosage. A certain amount of time is required for the cumulative thermal effect to be therapeutic. The duration of moxibustion is an important component of moxibustion dosage. A review of moxibustion therapy recommends 40-min suspended moxibustion sessions for optimal results (Lin et al., 2024), supported by an animal study showing HRV improvements after 20-min applications (Shu et al., 2017). Our abbreviated 15-min protocol may explain the lack of HRV response; insufficient duration may be an important reason for the lack of manifestation of moxibustion’s effect on HRV regulation.

We observed that the autonomic regulatory effect and stress-relieving effect of CV12 acupuncture, as well as the HR regulatory effects of both acupuncture and moxibustion, persisted for 5–10 min post-intervention. Previous studies have shown sustained effects of acupuncture and moxibustion in the treatment of various diseases (Leung et al., 2022; Yin et al., 2022; Wang et al., 2020). Research on acupuncture for depression has even found that the sustained effects of acupuncture are stronger and more extensive than the immediate effects (Wei et al., 2021). The sustained effects of acupuncture may serve as an important basis for achieving long-term therapeutic efficacy in the treatment of various diseases. The sustained effects of acupuncture on ANS regulation may facilitate long-term therapeutic outcomes by addressing ANS dysfunction in various related conditions, such as migraine (Li et al., 2020) and hypertension (Moreira et al., 2023). This is of particular importance for the long-term maintenance of human health and the prevention and treatment of chronic disease. We also observed that the therapeutic effects began to diminish 5–10 min post-intervention, which may partially explain why clinical acupuncture treatment requires multiple sessions and course-based administration. The mechanism by which single-session effects accumulate into long-term effects warrants further investigation.

Acupoints are the core of acupuncture therapy. We observed that the intervention effects of ST36 and CV12 on human ANS were largely consistent, but CV12 demonstrated more pronounced sustained effects, primarily manifested as prolonged enhancement of vagal nerve activity. ST36 is located in the lower leg and CV12 in the abdomen, and the different anatomical structures of the two points may be one of the reasons for the discrepancy. Both the vagus-adrenal axis and the sympathetic pathway can be regulated via specific autonomic pathways by stimulating specific acupoints (Liu et al., 2020). Researches indicate that the signaling of acupoints involves multiple pathways, and different sites and different intensities may activate different pathways to exert different autonomic regulatory effects (Liu et al., 2024; Yu et al., 2024). These findings suggest the potential of acupuncture therapy in regulating ANS branches, though how to achieve precise modulation through different acupuncture protocols remains to be explored.

Our study found that acupuncture reduces physiological stress, which is consistent with the results of two acupuncture studies (May and Bennett, 2023; Guo et al., 2024). Stress can be caused by physical, chemical and biological stimuli (such as work fatigue, illness and aging) as well as from internal sources of stressful information (such as work, academic pressure and emotional stress). Excessive and chronic stress can be detrimental to human health, with physical therapy currently being used as a primary tool. For example, practices such as controlled breathing (Balban et al., 2023), laughter therapy (Akimbekov and Razzaque, 2021), and mindfulness-based practices and contact with nature (Menardo et al., 2022) are often considered forms of psychological intervention, and have limitations due to their subjective nature. Exercise therapies such as yoga (R et al., 2023) require long-term adherence and have high patient compliance requirements. The limitations of such studies are evident in the data collection, primarily due to their reliance on self-reported scales that lack objective metrics. Stress triggers a neurobiological response, resulting in increased sympathetic nervous system activity, increased HR (Seddon et al., 2020), nd HRV variability. These symptoms serve as objective indicators of stress levels in the body. Research has shown that higher PSI is often associated with lower HRV and higher sympathetic activity, suggesting that the body is in a higher state of stress (Kim et al., 2018; Jiryis et al., 2022). Low HRV reduces the body’s ability to respond to internal and external stress. Acupuncture increases the overall activity of the vagus and autonomic nerves and reduces PSI in real time with sustained effects, suggesting that acupuncture has a positive anti-stress effect and that its mechanism is related to autonomic regulation.

Although no autonomic modulation effects were detected with moxibustion at ST36, we also observed a decrease in HR, consistent with the results of two previous animal studies (Li et al., 2021; Liu et al., 2019). In addition to nerves, the heart is also regulated by body fluids; adrenaline in the circulation speeds up the heart rate, while norepinephrine (NE) slows it down. Research has shown that navel moxibustion can increase NE levels in the hippocampus, prefrontal cortex and hypothalamus of rats in a stress model (Pan et al., 2023). Humoral factors may represent a direction for exploring the mechanism by which moxibustion reduces HR.

Our study shows that acupuncture can alleviate physical stress by regulating ANS activity, with distinct modulatory effects observed across different acupoints, confirming its beneficial role in modulating ANS function and stress response in healthy adults, and indicating potential applications in stress management and health maintenance. Further research is needed to determine how to activate different pathways through acupoint and acupuncture method selection to achieve different ANS regulatory effects. Similarly, moxibustion demonstrates marked efficacy in reducing HR, and the optimal moxibustion doses to achieve the desired ANS regulatory effect requires further investigation.

There are also limitations to this study. First, the study employed a self-comparison design without a control group, which precludes causal inference. Second, the duration of moxibustion in this study may have been insufficient. Additionally, the short follow-up period limits the assessment of long-term treatment effects.

## Data Availability

The raw data supporting the conclusions of this article will be made available by the authors, without undue reservation.

## References

[ref1] Agorastos A. Chrousos G. P. (2022). The neuroendocrinology of stress: the stress-related continuum of chronic disease development. Mol. Psychiatry 27, 502–513. doi: 10.1038/s41380-021-01224-9, PMID: 34290370

[ref2] Akimbekov N. S. Razzaque M. S. (2021). Laughter therapy: A humor-induced hormonal intervention to reduce stress and anxiety. Curr Res Physiol 4, 135–138. doi: 10.1016/j.crphys.2021.04.002, PMID: 34642668 PMC8496883

[ref3] Arakaki X. Arechavala R. J. Choy E. H. Bautista J. Bliss B. Molloy C. . (2023). The connection between heart rate variability (HRV), neurological health, and cognition: A literature review. Front. Neurosci. 17:1055445. doi: 10.3389/fnins.2023.1055445, PMID: 36937689 PMC10014754

[ref4] Balban M. Y. Neri E. Kogon M. M. Weed L. Nouriani B. Jo B. . (2023). Brief structured respiration practices enhance mood and reduce physiological arousal. Cell Rep. Med. 4:100895. doi: 10.1016/j.xcrm.2022.100895, PMID: 36630953 PMC9873947

[ref5] Birch S. Bovey M. Alraek T. Robinson N. Kim T. Lee M. (2020). Acupuncture as a treatment within integrative health for palliative care: A brief narrative review of evidence and recommendations. J. Altern. Complement. Med. 26, 784–791. doi: 10.1089/acm.2020.0032, PMID: 32924554

[ref6] Fan A. Y. (2023). Anti-inflammatory mechanism of electroacupuncture involves the modulation of multiple systems, levels and targets and is not limited to "driving the vagus-adrenal axis". J. Integr. Med. 21, 320–323. doi: 10.1016/j.joim.2023.06.001, PMID: 37331861

[ref7] Fleckenstein J. Kruger P. Ittner K. (2018). Effects of single-point acupuncture (HT7) in the prevention of test anxiety: results of a RCT. PLoS One 13:e0202659. doi: 10.1371/journal.pone.0202659, PMID: 30161153 PMC6116988

[ref8] Fu Z. T. Liu C. Z. Kim M. R. Liu Y. D. Wang Y. Fu Y. M. . (2024). Acupuncture improves the symptoms, serum ghrelin, and autonomic nervous system of patients with postprandial distress syndrome: a randomized controlled trial. Chin. Med. 19:162. doi: 10.1186/s13020-024-01028-3, PMID: 39568071 PMC11580632

[ref9] Goreis A. Prillinger K. Bedus C. Lipp R. Mayer A. Nater U. M. . (2023). Physiological stress reactivity and self-harm: A meta-analysis. Psychoneuroendocrinology 158:106406. doi: 10.1016/j.psyneuen.2023.106406, PMID: 37783020

[ref10] Gosain R. Gage-Bouchard E. Ambrosone C. Repasky E. Gandhi S. (2020). Stress reduction strategies in breast cancer: review of pharmacologic and non-pharmacologic based strategies. Semin. Immunopathol. 42, 719–734. doi: 10.1007/s00281-020-00815-y 32948909 PMC7704484

[ref11] Guo Z. Ren Z. Yao J. Li Y. Che Z. Yu Z. . (2024). Does acupuncture treatment modulate inflammatory cytokines in rodent models of depression? A systematic review and meta-analysis. Front. Behav. Neurosci. 18:1329638. doi: 10.3389/fnbeh.2024.1329638, PMID: 38292326 PMC10823433

[ref12] Hamvas S. Hegyi P. Kiss S. Lohner S. Mcqueen D. Havasi M. (2023). Acupuncture increases parasympathetic tone, modulating HRV - systematic review and meta-analysis. Complement. Ther. Med. 72:102905. doi: 10.1016/j.ctim.2022.102905, PMID: 36494036

[ref13] Hill D. Conner M. Clancy F. Moss R. Wilding S. Bristow M. . (2022). Stress and eating behaviours in healthy adults: a systematic review and meta-analysis. Health Psychol. Rev. 16, 280–304. doi: 10.1080/17437199.2021.1923406, PMID: 33913377

[ref14] Jie L. Xie Y. Xu Z. (2018). Analytical study on the patterns of clinical application of Zusanli (ST36) in the compendium of acupuncture and moxibustion. Global Traditional Chinese Med 9, 1398–1401. doi: 10.3969/j.issn.1674-1749.2018.09.018

[ref15] Jiryis T. Magal N. Fructher E. Hertz U. Admon R. (2022). Resting-state heart rate variability (HRV) mediates the association between perceived chronic stress and ambiguity avoidance. Sci. Rep. 12:17645. doi: 10.1038/s41598-022-22584-4, PMID: 36271286 PMC9587282

[ref16] Khan A. A. Lip G. Y. H. Shantsila A. (2019). Heart rate variability in atrial fibrillation: the balance between sympathetic and parasympathetic nervous system. Eur. J. Clin. Investig. 49:e13174. doi: 10.1111/eci.13174, PMID: 31560809

[ref17] Kim H. G. Cheon E. J. Bai D. S. Lee Y. H. Koo B. H. (2018). Stress and heart rate variability: A Meta-analysis and review of the literature. Psychiatry Investig. 15, 235–245. doi: 10.30773/pi.2017.08.17, PMID: 29486547 PMC5900369

[ref18] Kouzuma N. Taguchi T. Higuchi M. (2022). Heart rate and autonomic nervous system activity relationship during acupuncture associated with postural change and effect on menopausal symptoms: a prospective randomized trial. Med. Acupunct. 34, 299–307. doi: 10.1089/acu.2022.0004, PMID: 36311889 PMC9595640

[ref19] Leung K. Ma O. C. Qin Z. Ting H. Lau A. H. Lun K. K. . (2022). Acupuncture for de Quervain's tenosynovitis: a randomized controlled trial. Phytomedicine 104:154254. doi: 10.1016/j.phymed.2022.154254, PMID: 35728386

[ref20] Li Q. Wang W. Ma Q. Xia R. Gao B. Zhu G. . (2021). Moxibustion improves chronic heart failure by inhibiting autophagy and inflammation via upregulation of mTOR expression. Evid. Based Complement. Alternat. Med. 2021:6635876. doi: 10.1155/2021/6635876 33603819 PMC7872756

[ref21] Li Y. X. Xiao X. L. Zhong D. L. Luo L. J. Yang H. Zhou J. . (2020). Effectiveness and safety of acupuncture for migraine: an overview of systematic reviews. Pain Res. Manag. 2020:3825617. doi: 10.1155/2020/3825617, PMID: 32269669 PMC7125485

[ref22] Lin X. Y. Zhang S. A. Xu Y. H. Yang Y. Wang J. (2024). Research progress on influencing factors affecting the efficacy of moxibustion in treating knee osteoarthritis. Acupuncture Research 49, 185–191. doi: 10.13702/j.1000-0607.20221093, PMID: 38413040

[ref23] Liu Z. H. Huang J. L. Yan D. S. Liang S. Zhao S. T. Zhang M. Z. . (2024). Effect of “needle sensation” and the real-time changes in autonomic nervous system activity during acupuncture analgesia. Front. Neurosci. 18:1349059. doi: 10.3389/fnins.2024.1349059, PMID: 38560046 PMC10979699

[ref24] Liu N. N. Jia X. Z. wang J. Zhu G. Q. Li D. Li Q. L. . (2019). Moxibustion improves cardiac function by up-regulating autophagy-related proteins of cardiomyocytes in rats with chronic heart failure. Zhen Ci Yan Jiu 44, 25–30. doi: 10.13702/j.1000-0607.170968, PMID: 30773858

[ref25] Liu S. Wang Z. F. Su Y. S. Ray R. S. Jing X. H. Wang Y. Q. . (2020). Somatotopic organization and intensity dependence in driving distinct NPY-expressing sympathetic pathways by Electroacupuncture. Neuron 108, 436–450.e7. doi: 10.1016/j.neuron.2020.07.015, PMID: 32791039 PMC7666081

[ref26] Lu S. S. Wang B. Wang J. Q. Guo Y. Li S. S. Zhao S. H. . (2023). Moxibustion for the treatment of cancer and its complications: efficacies and mechanisms. Integr. Cancer Ther. 22, 1–18. doi: 10.1177/15347354231198089 PMC10521285 37746720

[ref27] May N. Bennett A. (2023). The impact of acupuncture on self-perceived stress and ADHD core symptomatology in an adult, atomoxetine-taking ADHD participant. Insights from an In-depth Single Case Study. Integr Med (Encinitas) 22, 28–36, PMID: 37534023 PMC10393382

[ref28] Meira Do Valle S. S. Hong H. (2024). Acupuncture treatment for generalized anxiety disorder by activating the vagus nerve and improving heart-rate variability and heart-rhythm coherence, a case-series study. Med. Acupunct. 36, 21–26. doi: 10.1089/acu.2023.0036, PMID: 38405597 PMC10890946

[ref29] Menardo E. Di Marco D. Ramos S. Brondino M. Arenas A. Costa P. . (2022). Nature and mindfulness to cope with work-related stress: A narrative review. Int. J. Environ. Res. Public Health 19:5948. doi: 10.3390/ijerph19105948, PMID: 35627491 PMC9140663

[ref30] Moreira R. M. Ros Rio R. C. Boggiss É A. Lima R. A. Silva P. A. Silva K. P. D. . (2023). Effect of systemic and auricular acupuncture with a 2/100 Hz frequency and Nogier frequency in fibromyalgia: a randomized clinical trial, pilot study. J. Acupunct. Meridian Stud. 16, 139–151. doi: 10.51507/j.jams.2023.16.4.139 37609769

[ref31] Pan M. M. Wang Q. Y. Hou J. L. Zhang T. Jiang Y. Yang L. P. (2023). Effects of umbilical moxibustion on phobic behavior and monoamine neurotransmitters in stress-model rats. Zhongguo Zhen Jiu 43, 191–196. doi: 10.13703/j.0255-2930.20211123-k0004, PMID: 36808514

[ref32] Perulli M. Scala I. Venditti R. Amadio A. Luigia Gambardella M. Quintiliani M. . (2023). Short- vs long-term assessment of heart rate variability: clinical significance in Dravet syndrome. Epilepsy Behav. 146:109357. doi: 10.1016/j.yebeh.2023.109357, PMID: 37499580

[ref33] Pluntke U. Gerke S. Sridhar A. Weiss J. Michel B. (2019). Evaluation and classification of physical and psychological stress in firefighters using heart rate variability. Annu Int Conf IEEE Eng Med Biol Soc 2019, 2207–2212. doi: 10.1109/EMBC.2019.8856596 31946339

[ref34] Qu M. Chen Y. Yang D. He M. Lin X. (2024). Research progress on nerve pathways of acupuncture at Zhongwan to regulate gastric motility. Global Traditional Chinese Medicine 17, 337–344. doi: 10.3969/j.issn.1674-1749.2024.02.031

[ref35] R P. Kumar A. P. Dhamodhini K. S. Venugopal V. Silambanan S. K M. . (2023). Role of yoga in stress management and implications in major depression disorder. J. Ayurveda Integr. Med. 14:100767. doi: 10.1016/j.jaim.2023.100767 37741161 PMC10520539

[ref36] Schleinzer A. Moosburner A. Anheyer D. Burgahn L. Cramer H. (2024). Effects of yoga on stress in stressed adults: a systematic review and meta-analysis. Front. Psych. 15:1437902. doi: 10.3389/fpsyt.2024.1437902, PMID: 39553891 PMC11563964

[ref37] Seddon J. A. Rodriguez V. J. Provencher Y. Raftery-Helmer J. Hersh J. Labelle P. R. . (2020). Meta-analysis of the effectiveness of the Trier social stress test in eliciting physiological stress responses in children and adolescents. Psychoneuroendocrinology 116:104582. doi: 10.1016/j.psyneuen.2020.104582, PMID: 32305745

[ref38] Shaffer F. Ginsberg J. P. (2017). An overview of heart rate variability metrics and norms. Front. Public Health 5:258. doi: 10.3389/fpubh.2017.00258, PMID: 29034226 PMC5624990

[ref39] Shu Q. Sun D. H. Wang H. Liang F. X. Gerhard L. Daniela L. . (2017). Differences of acupuncture and moxibustion heart rate variability in qi-deficiency syndrome: a randomized controlled trial. Chin. Acupunct. Moxibustion 37, 25–30. doi: 10.13703/j.0255-2930.2017.01.006 29231318

[ref40] Song Y. J. Liang F. X. Wang H. Gerhard L. Wu S. Li J. . (2020). Immediate effect of acupuncture and moxibustion at Guanyuan (CV 4) and Zusanli (ST 36) on heart rate variability in patients with qi deficiency syndrome. Zhongguo Zhen Jiu 40, 1047–1051. doi: 10.13703/j.0255-2930.20190727-k0003, PMID: 33068344

[ref41] Thielmann B. Pohl R. Bockelmann I. (2021). Heart rate variability as a strain indicator for psychological stress for emergency physicians during work and alert intervention: a systematic review. J Occup Med Toxicol 16:24. doi: 10.1186/s12995-021-00313-3, PMID: 34187497 PMC8240085

[ref42] Wang W. J. Lu J. Niu C. S. Huang Y. R. Ma Q. A Y. G. . (2010). Effects of electroacupuncture of unilateral and bilateral "zusanli" (ST 36) on serum TNF-alpha, IL-1 and IL-4 levels in rats with chronic inflammatory pain. Zhen Ci Yan Jiu 35, 429–432. 21375016

[ref43] Wang F. Yue Z. (2021). Technique of acupuncture and Moxibustion. Beijing: China Traditional Chinese Medicine Press.

[ref44] Wang L. L. Zhu J. Y. Ren Z. X. Zhang H. L. Wu Y. R. (2020). Observation on therapeutic effect of electroacpuncture combined with penetrating moxibustion for postpartum pelvic organ prolapse. Zhongguo Zhen Jiu 40, 157–161. doi: 10.13703/j.0255-2930.20190210-00014, PMID: 32100501

[ref45] Wei X. Y. Chen H. Guo C. Tan W. L. Zhan S. H. (2021). The instant and sustained effect of Electroacupuncture in postgraduate students with depression: an fMRI study. Neuropsychiatr. Dis. Treat. 17, 873–883. doi: 10.2147/NDT.S307083, PMID: 33776442 PMC7989050

[ref46] Yan S. Xiong Z. Liu X. Liu C. Liu B. (2022). Review of clinical research in acupuncture and moxibustion from 2010 to 2020 and future prospects. Zhongguo Zhen Jiu 42, 116–118. doi: 10.13703/j.0255-2930.20210331-0008, PMID: 35025168

[ref47] Yang S. Wu Y. R. Zhan Z. Pan Y. H. Jiang J. F. (2024). State- and frequency-dependence in autonomic rebalance mediated by intradermal auricular electroacupuncture stimulation. Front. Neurosci. 18:1367266. doi: 10.3389/fnins.2024.1367266, PMID: 38846714 PMC11153749

[ref48] Yin X. Li W. Liang T. Lu B. Yue H. Li S. . (2022). Effect of electroacupuncture on insomnia in patients with depression: a randomized clinical trial. JAMA Netw. Open 5:e2220563. doi: 10.1001/jamanetworkopen.2022.20563, PMID: 35797047 PMC9264041

[ref49] You J. Ye J. Li H. Ye W. Hong E. (2021). Moxibustion for chronic fatigue syndrome: a systematic review and meta-analysis. Evid. Based Complement. Alternat. Med. 2021:6418217. doi: 10.1155/2021/6418217, PMID: 34804182 PMC8601810

[ref50] Yu Z. Lu M. J. Han X. Xu B. (2024). Effect characteristics of electroacupuncture at the acupoints of different regions in regulating gastric motility of the rats: response time. Chinese Acup. Moxibustion 44, 73–77. doi: 10.13703/j.0255-2930.20220409-0002, PMID: 38191163

